# Gut microbiota and circadian rhythm in Alzheimer’s disease pathophysiology: a review and hypothesis on their association

**DOI:** 10.1038/s41514-023-00104-6

**Published:** 2023-05-02

**Authors:** Mohammad Rafi Khezri, Morteza Ghasemnejad-Berenji

**Affiliations:** 1 Urmia University of Medical Sciences, Faculty of Pharmacy, Urmia, Iran; 2 Department of Pharmacology and Toxicology, Faculty of Pharmacy, Urmia University of Medical Sciences, Urmia, Iran; 3 Research Center for Experimental and Applied Pharmaceutical Sciences, Urmia University of Medical Sciences, Urmia, Iran

**Keywords:** Microbiology, Neuroscience

## Abstract

Alzheimer’s disease (AD) is the most common neurodegenerative disease and the leading cause of dementia worldwide. Different pathologic changes have been introduced to be involved in its progression. Although amyloid-β (Aβ) deposition and tau hyperphosphorylation and aggregation are mainly considered the main characterizations of AD, several other processes are involved. In recent years, several other changes, including alterations in gut microbiota proportion and circadian rhythms, have been noticed due to their role in AD progression. However, the exact mechanism indicating the association between circadian rhythms and gut microbiota abundance has not been investigated yet. This paper aims to review the role of gut microbiota and circadian rhythm in AD pathophysiology and introduces a hypothesis to explain their association.

## Alzheimer’s disease: an overview

Alzheimer’s disease (AD) is the most common neurodegenerative disorder and the leading cause of dementia worldwide^1^. AD patients experience a variety of symptoms, including memory loss, cognitive alterations, and behavioral changes^2,3^. AD-related dementia is linked to neurodegeneration, initially characterized by synaptic and neuronal loss^4^. These processes are accompanied by microglial cell proliferation^5,6^, astrogliosis^7^, and the presence of neurofibrillary tangles composed of hyperphosphorylated tau and dystrophic neurites^8,9^. More recent studies provide uncovered evidence, suggesting that another component of neurodegenerative AD includes the possibility of interference with the process of adult hippocampus neurogenesis^10,11^. Additionally, studies in transgenic animal models of AD have shown a marked aberrant process of adult neurogenesis in the hippocampus^12–14^.

Regarding the various neuropathological features of AD, cognitive alterations in AD patients are associated with synaptic injury in the limbic system and neocortex^15,16^. However, the other main characterization of AD is the progressive deposition of amyloid-β (Aβ) protein which has been linked to mentioned neuropathological changes^17,18^. Abnormal Aβ accumulation is caused by a disturbance in the imbalance between Aβ production, aggregation, and clearance. Clearance of Aβ is mediated by proteolytic enzymes, such as chaperone molecules such as apoE^19^, neprilysin^20^, lysosomal (e.g., autophagy)^21^, and non-lysosomal routes (e.g., proteasome)^22^. There are two main forms of AD, including familial and sporadic forms. Although in familial forms of AD, mutations cause an increase in Aβ production or aggregation, in sporadic AD, alterations of the clearance pathways might play a crucial role^23^. Accumulation of Aβ leads to the generation of Aβ oligomers and fibrils, which are the main components of the Aβ plaques^24^. However, most evidence suggests that the Aβ oligomers rather than the fibrils mediate the Aβ-induced synapto-toxicity^25,26^.

Axonal pathology and synaptic loss are probably the key neuropathological changes leading to dementia in AD^27–29^; however, other factors have been identified to be involved. In recent years, new studies have suggested several other mechanisms in other organs involved in the pathophysiology of AD. In this regard, changes in intestinal microbiota have been closely linked to the progression of AD, which will be discussed in the next sections.

## Gut microbiota and Alzheimer’s disease

There are billions of colonized microbes in the human gut. Increasing evidence suggests that there is a bidirectional association between the human gut microbiota and the brain, which is known as Microbiota–Gut–Brain Axis (MGBA)^30^. Gut dysbiosis has been associated with a variety of diseases, especially neurological conditions such as neurodegenerative diseases^31,32^. In this regard, experimental studies have revealed that gut flora is involved in the regulation of brain functions such as memory and learning^33^. More importantly, the function and composition of intestinal flora affect the pathophysiology of age-related cognitive impairment and dementia, suggesting its crucial role in the onset and progression of AD^34–36^.

### Gut microbiota and brain function

The association between the gut flora and the Central nervous system (CNS) is due to the interaction between the intestine and the brain with each other via the nervous system or chemicals which cross the blood-brain barrier (BBB)^37^. The gut flora produces chemical substances (i.e., amino acids and monoamines) that reach the neurons of CNS via the vascular and lymphatic system and can affect their activity, with probable influences on behavior^38^. On the other hand, the gut microbiota is affected by the messages as neurotransmitters sent by the brain^39,40^. Several communication pathways between the brain and gut have been investigated^41^. The Vagus nerve plays a central role in the connection between the gut and the autonomic nervous system^42^. This nerve ends to the brain stem nuclei, which give efferent fibers and receive afferent fibers. In this pathway, stem nuclei may regulate many gut activities and send signals to the other regions of the brain, such as the cortical areas and thalamus^43^. Additionally, the enteric nervous system can send and receive signals from the CNS via the gut flora^44^. Also, blood circulation is involved in the exchange between the gut and brain^45^. Intestinal mucosa and BBB allow the passage of endocrine and immune molecules, the most important of which are hormones and cytokines, which can affect the function of both the gut and brain^46^. Interestingly, it has been reported that gut microbiota affects the maturation of the endocrine, immune, and nervous system in germ-free mice^43^. Gut microbiota regulates MGBA through different routes. For instance, these microorganisms can synthesize and release neuromodulators and neurotransmitters, such as biogenic amines (e.g., histamine, serotonin, and dopamine), short-chain fatty acids (SCFAs), and other metabolites produced from amino acids such as GABA or serotonin and tryptophan^41^. On the other hand, the other possibility for MGBA regulation by gut bacteria is that these microorganisms produce substances that are toxic to the brain, such as ammonia and D-lactic acid^47^. In addition, during several inflammatory processes, the gut microbiota produces and releases other toxic proteins to the brain, such as host innate immune activators^48^. Alterations in the mentioned processes, especially immunological processes, can contribute to anxiety, memory impairment, and other cognitive alterations^47,49,50^. Recent studies reported that these alterations are associated with a variety of neurological conditions, including depression^51^, drug-resistant epilepsy^52^, and neurodegenerative diseases, especially AD, Parkinson’s disease, and multiple sclerosis^53–55^.

### Alterations of the gut microbiota in AD patients

Analysis of intestinal microbiota in AD patients was first conducted by Cattaneo et al.^56^. In this study, to evaluate the correlation between gut flora and cognitive impairment, the abundance of bacterial gut microbiota taxa in the feces of healthy controls, patients with cognitive impairment and brain amyloidosis, and patients with cognitive impairment without brain amyloidosis, as well as the levels of inflammatory mediators in their blood were measured. The results of this study revealed that the abundance of *Escherichia*/*Shigella* species as pro-inflammatory species were increased in the gut flora of patients with cognitive impairment and brain amyloidosis. In contrast, a decrement in the abundance of anti-inflammatory species *Eubacterium rectale* was detected. Additionally, their results showed a significant correlation between the abundance of gut flora and levels of pro-inflammatory factors IL-1β, NLRP3, and CXCL2. In another study, the proportion of different intestinal microbiota species in AD patients compared to age and sex matched the healthy controls^57^. Their results showed that the abundance of bacteria with the ability of butyrate synthesis in the flora of AD patients’ feces was reduced. Additionally, they found that fecal samples from AD patients induce lower expression of p-glycoprotein as a key regulator of intestinal homeostasis in epithelial cells of the intestine. There are several other interesting results indicating the association between gut flora and biochemical parameters in blood samples from AD patients. One of these works shows that adiponectin levels correlate with *Faecalibacterium*, Acidimicrobiia, Oscillospiraceae, Actinobacteria, *Prevotella*, and Christensenellaceae R-7. Also, Acidobacteriota is linked to total bilirubin, while Firmicutes, Castellaniella alcaligenes, Acidobacteriales bacterium, Lachnospiraceae, *Klebsiella pneumoniae*, and Christensenellaceae correlate with the level of CRP in the blood of patients with AD^58^. Additionally, another study revealed that patients with AD or mild cognitive impairment show an increase in bacterial taxa, including Erysipelotrichales, Erysipelatoclostridiaceae, Patescibacteria, Saccharimonadia, and Saccharimonadales, compared with normal control subjects, which were positively associated with APOE 4, and negatively correlated with memory^59^.

### Gut flora and AD-related pathophysiology

Neuroinflammation plays a crucial role in the progression of AD^40^. However, recent findings suggest a close association between intestinal microbiota and neuroinflammation^60^. *Bacteroidetes* family of Gram-negative bacteria, which constitute a considerable abundance of the gut microbiota, releases a mixture of neurotoxins, mainly pro-inflammatory lipopolysaccharides (LPS), leading to trigger systemic inflammation via the promotion of pro-inflammatory cytokines production^61^. It has been reported that LPS levels in specimens from AD patients elevated by three times in the hippocampus and two times in the neocortex when compared to healthy controls^62,63^. It has been elucidated that LPS injection during the development of the brain induces microglial activity leading to elevated levels of the pro-inflammatory cytokines IL-6, IL-1β, and TNF-α^64^. The association between LPS and AD pathology has been linked to its ability to initiate amyloid fibrillogenesis in co-incubation with Aβ peptide^65^. Additionally, it has been reported that systemic injection of LPS contributes to Aβ deposition and tau aggregation in APPswe transgenic mice^66^. Interestingly, Gram-negative *Escherichia coli*-derived LPS and fragments have been detected in Aβ plaques from AD patients^67^. On the other hand, gut flora can regulate the levels of microRNAs (miRs), a group of important factors in AD pathophysiology. In this regard, it has been reported that gut bacteria-derived LPS can affect miR levels in AD^68^. Additionally, it has been demonstrated that the neurotropic herpes simplex virus-1 and Gram-negative bacteria *Bacteroides fragilis* share a final common pathway of NF-κB and microRNA-146a induction which results in the stimulation of neuroinflammatory pathways^69^. In another study, *Bacteroides fragilis*-derived LPS has been shown to cross the BBB into brain-parenchyma and neuronal-cytoplasm through systemic circulation and leads to induce the expression of pro-inflammatory miRs, miR- 146a and miR-155, introduced as a contributor to the onset of AD^70^.

Regardless of the neuroinflammation, the gut microbiota is involved in the other aspects of AD pathophysiology. Animal studies revealed a correlation between *Akkermansia* and seven other bacterial genera with the cerebral soluble Aβ42 levels^71^. *Butyricicoccus* and *Akkermansia*, two main regulators of gut barrier integrity^72^, have been shown to be negatively associated with the levels of pathogenic Aβ42 in the brain. It has been hypothesized that a reduced proportion of these bacteria in gut flora may lead to the LPS influx into the brain in AD, as a leaky gut has been detected in patients with AD^73,74^. Therefore, an increased proportion of *Bacteroides*, along with a reduced abundance of *Butyricicoccus* and *Akkermansia*, may result in elevated LPS translocation from the intestines into the brain via systemic circulation^74^.

## BMAL1 and Alzheimer’s disease

**Fig. 1:**
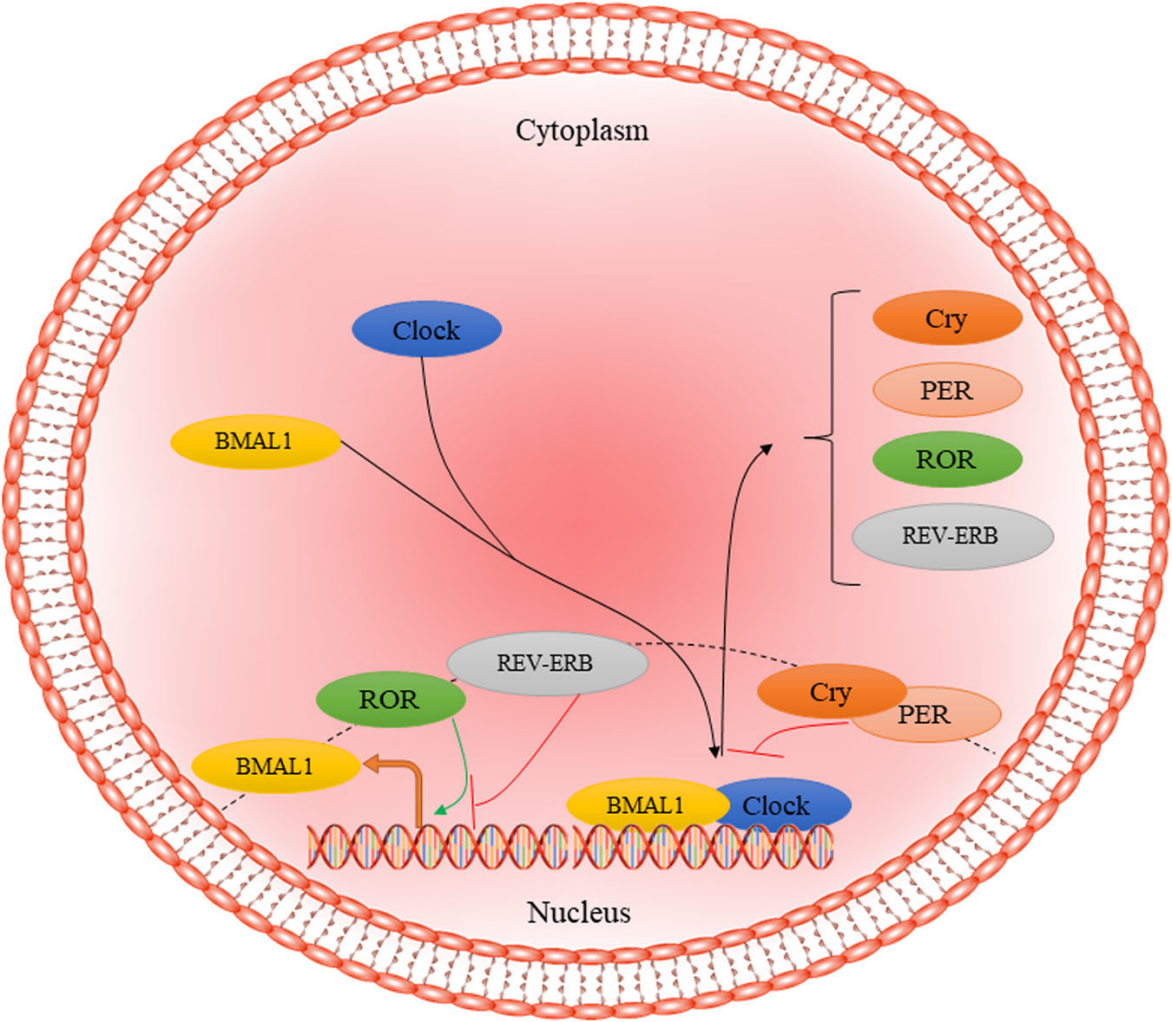
Regulators of circadian rhythm in mammalian cells. CLOCK-BMAL1 complex translocates to the nucleus and binds to E-box elements to activate the expression of Cry, PER, RPR, and REV-ERB as the negative regulators of primary complex activity. CRY1/2 cryptochrome, PER period, ROR retinoid-related orphan receptor, REV-ERB reverse erythroblastosis virus α.

Altered circadian rhythms, irrespective of cause, have been implicated in a multitude of diseases, including metabolic diseases such as obesity^75,76^, sleep disorders^77^, psychiatric disorders such as bipolar illness^78^, and neurodegenerative diseases such as AD^79^. Brain and muscle Arnt-like protein-1 (BMAL1), encoded by the *ARNTL* gene, is a core regulator of the circadian clock in humans and is known as the only irreplaceable clock factor regulating rhythmic behaviors^80,81^. At the molecular inspection, BMAL1 regulates nearly 24 h autonomous circadian oscillations through the transcriptional–translational feedback loop (TTFL). Circadian locomotor output cycles kaput (CLOCK) together with BMAL1 dimerize and bind to the E-box motifs form the positive limb leading to express the cryptochrome (CRY1/2), period (PER1/2/3), retinoid-related orphan receptor-α (RORα), and reverse erythroblastosis virus α (REV-ERBα). Finally, CRY and PER proteins interact with each other, forming cytoplasmic heterodimers, which translocate to the nucleus to inhibit the positive limb expression (Fig. 1)^82^. On the other hand, RORα and REV-ERBα restrain and facilitate the expression of BMAL1, respectively^83^. In humans, all fully differentiated cells have this molecular clock based on circadian rhythmicity^84^. The circadian system is involved in the regulation of different physiological processes, such as the rest-activity cycle, food-intake behavior, and glucose metabolism^85^. In addition to the circadian system, BMAL1 regulates other aspects of cell survival, such as oxidative response and redox homeostasis, along with nuclear factor-related factor 2 (Nrf2), through regulating the rhythmic expression of Prdx6^86,87^. Further, BMAL1 is involved in regulating inflammatory processes^88^ and sensitivity to insulin^89^. It has been reported that *ARNTL* disruption leads to the progression of aging-related diseases, such as type 2 diabetes mellitus and neurodegenerative diseases^90,91^.

One of the most common symptoms of AD is a disrupted circadian rhythm, characterized by awakening and increased sleep during the night and day, respectively^92^. AD-related loss of the normal circadian rhythm has been widely associated with altered BMAL1 activity^93^ as aberrant BMAL11 activity and levels have been detected in samples from AD patients. In this regard, it has been demonstrated that patients with AD show a higher prevalence of T carriers in BMAL1 rs.2278749 T/C in comparison with healthy controls in whole blood samples from the antecubital vein^94^. In addition, different levels of BMAL1 levels from AD patients and healthy controls have been detected in the occipital cortex, frontal cortex, temporal cortex, pineal glands, and parietal cortex^95,96^. In a molecular inspection, excessive studies have shown a correlation between BMAL1 and different factors involved in the pathophysiology of AD. In this regard, mutual communication between BMAL1 and Aβ deposition has been reported in different studies. The aberrant expression of BMAL1 protein has been proposed to be a result of the Aβ effect in mouse hippocampus^97^. Additionally, it has been shown that Aβ can accelerate BMAL1 degradation in an animal model of AD^98^. On the other hand, the BMAL1 loss has been shown to accelerate the Aβ plaques accumulation^99^. Additionally, BMAL1 is associated with responses of astrocytes and microglia to Aβ deposits. It has been shown that *BMAL1* deletion in mice contributes to exacerbated astrocyte activation around Aβ plaques along with altered gene expression^100^. Also, REV-ERBs inhibition has been shown to increase BMAL1 transcription and induce microglial Aβ phagocytic activity leading to enhance Aβ clearance in the 5XFAD mouse model of AD^101^.

In addition to the mentioned points, altered BMAL1 in astrocytes in different studies revealed several pathological changes that promote AD. In a study, lower cortactin expression, lower Rho-GTP levels, and impaired actin cytoskeleton dynamics were observed in BMAL1^−/−^ astrocytes leading to disrupted synaptic integrity. Also, in this study, BMAL1^−/−^ mice showed a significant decrement of synaptic coverage by astrocytes, which is associated with chronodisruption-induced cognitive deficit^102^. On the other hand, BMAL1 regulates astrocyte-to-neuron communication via the prevention of GABA accumulation in intracellular space^100^. In this regard, it was shown that treatment of BMAL1cKO mice with GABA receptor antagonists led to abolished cognitive functions^103^. Further, deletion of astrocyte-specific BMAL1 led to neuronal death and astrogliosis, a hallmark of AD, leading to enhanced expression of inflammatory genes^104^. In addition to astrocytes, it has been reported that partial knockdown of BMAL1 in neurons results in impaired structure and dysfunction of synapses and spontaneous neurodegeneration^105^. There is no idea about the aberrant levels of BMAL1 in AD. In this regard, it can be referred to regulators of BMAL1 with altered activity in AD to explain this disruption. One of the main regulators of BMAL1 is Sirtuin 1 (SIRT1) involved in its regulation through PER2 deacetylation Lower expressions of SIRT1 in samples from AD patients has been detected^106,107^. Mitogen-activated protein kinase (MAPK), the other altered factor in AD, has been shown to negatively regulate BMAL1 via its phosphorylation at Thr-534^108^. The other regulator of BMAL1 is glycogen synthase kinase 3 (GSK-3), which plays a crucial role in the pathophysiology of AD via phosphorylation of tau protein^109,110^. This evidence shows that the disruption of the level of BMAL1 does not happen by itself in AD; as a secondary factor, it can be due to the disruption of its regulators.

## A hypothesis on the association between BMAL1, IgA, and intestinal microbiota in Alzheimer’s disease

Many studies reveal that the composition of gut microbiota oscillates rhythmically, mainly in response to several body rhythms such as circadian rhythm^111^. In a study on mice gut flora, it was reported that a relative abundance of Lactobacillales, Clostridiales, Bacteroidales, Firmicutes, Bacteroidetes, *Clostridium* spp, *Ruminococcaceae* spp, *Lachnospiraceae* spp, *Bacteroides*, *Anaeroplasma*, and *Lactobacillaceae* spp. rhythmically oscillate in a 24-h period, which may be regulated by different factors involved in circadian rhythm^111,112^. In addition to gut flora abundance, changes in its functionality have been reported in response to altered light-dark rhythmicity. In this case, it has been elucidated that in mice, which exhibit light-dark rhythmicity opposite to humans, gut flora favored energy metabolism, cell growth, and DNA repair during the dark phase of maximum activity^111^.

Although many studies have shown the association between BMAL1 and AD, most of these studies have dealt with this connection in the CNS. However, the interaction between BMAL1 and gut microbiota has been reported in several studies. In this case, Zhang and colleagues have examined the effect of intermittent photoperiod on gut microbiota abundance in mice^113^. Their results reveal that intermittent photoperiod (16 [3 h-L/1 h-D]: 8 D) leads to enhance the circadian rhythms of c BMAL1, cBmal2, cCry1, and cCry2 in the hypothalamus and increases the expression of cClock, c BMAL1, and cCry2 in the liver and seven clock genes in the cecal wall. Additionally, they found that these changes eventually resulted in altered composition and metabolic function of the cecal microbiota in a way that the concentrations of SCFAs and the abundance of SCFA-producing genera, such as *Odoribacter*, significantly increased under the intermittent photoperiod treatment. To explain how circadian rhythm regulators, mainly BMAL1, regulate the abundance and function of gut flora, a recent study by Penny et al.^114^ showed an interesting result which can be discussed here. In this study, it was shown that the immunoglobulin A (IgA) secretion follows a rhythmic oscillation. Additionally, they found that this rhythmicity in IgA secretion influences gut microbiota abundance. The most interesting point in this study was the regulation of rhythmic IgA secretion and gut microbiota proportion by circadian rhythm, as detected, followed by the deletion of the ARNTL gene. These results may provide new insights into a more detailed description of the role of BMAL1 in AD. It can be said that disturbance of BMAL1 levels in AD followed by alterations in its regulators may lead to the lack of IgA rhythmic secretion and, eventually, altered microbiota abundance. To support these results, altered levels of IgA, as well as elevated numbers of IgA+ cells, have been detected in the cornu ammonis region of AD patients^115^, which may be due to disturbance of BMAL1 activity in these patients. This issue may explain the probable therapeutic role of intravenous immunoglobulin in AD^116^, while rhythmic administration of IgA may provide better results in this case. In addition to mentioned points, according to this possible communication, a mechanism can be proposed to explain the BBB disruption in AD. In this regard, it has been shown that disruption of BMAL1 is accompanied by impaired BBB and efflux transport^117,118^. On the other hand, it has been elucidated that an altered proportion of gut bacteria results in increased BBB leakage^119^, which may be linked to BMAL1-IgA communication. These processes may explain the therapeutic potential of melatonin on AD progression in different studies, as it has been shown that it modulates SIRT1, MAPK, and GSK-3 and induces BMAL1 activity^120^. Regardless of the role of these factors, Gao et al.^121^ found a link between melatonin and intestinal barrier dysfunction in mice. In this study, it was reported that reductions in melatonin levels followed by sleep deprivation contribute to increased pro-inflammatory cytokines, reduced anti-inflammatory cytokines, and colonic mucosal injury. They linked these changes to reduced gut flora diversity, decreased *Bacteroides, Akkermansia*, and *Faecalibacterium*, and increased *Aeromonas* resulting from reduced melatonin levels.

**Fig. 2:**
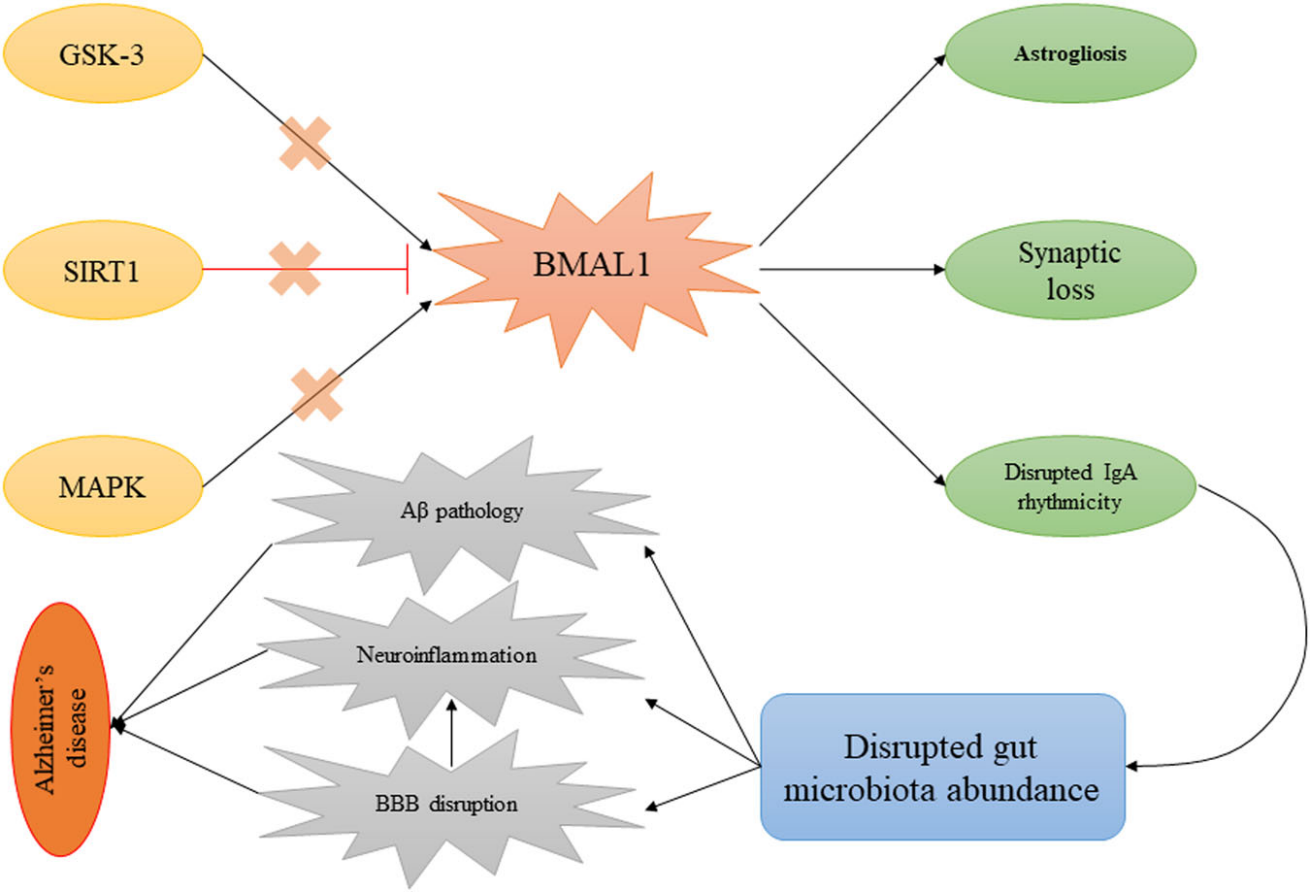
Gut-brain axis and circadian rhythm association. Aberrant activity of SIRT1, MAPK, and GSK-3 results in altered BMAL1 activity in AD. On the other hand, disruption in BMAL1 activities may contribute to altered IgA secretion and gut microbiota abundance. This process induced Aβ deposition, neuroinflammation, and BBB disruption leading to AD progression. AD Alzheimer’s disease, BMAL1 brain and muscle ARNT-Like 1, GSK-3 glycogen synthase kinase, MAPK mitogen-activated protein kinases, SIRT1 Sirtuin 1.

In addition to the role of BMAL1 in the regulation of gut microbiota abundance, the opposite association should also be examined, which can be considered as a regulatory loop between gut microbiota and BMAL1. In this regard, gut microbiota-derived chemicals are shown to be involved in the regulation of circadian rhythms via the regulation of BMAL1 expression. In a study by Leone et al., it was found that treatment of hepatic cells with sodium acetate and sodium butyrate changes the expression of BMAL1 and its regulator, PER2^122^. In addition, it was observed that treatment with SFCAs, especially butyrate, results in the induction of BMAL1 expression. Figure 2 depicts the hypothesized association between BMAL1 and IgA-gut microbiota in AD.

## Future perspective

The regulatory effects of BMAL1 on gut microbiota seem to be mediated by IgA. This connection may explain the results of recent findings on altered IgA levels in AD patients which can be due to aberrant activity of BMAL1. However, the association between BMAL1 with gut microbiota in any way could provide a therapeutic target to slow the progression of AD. Further studies may be required to find other possible mechanisms explaining the connection between BMAL1 and gut flora activity. In addition, altered oscillation secretion of IgA due to aberrant activity of BMAL1 can be compensated therapeutically to block the adverse effects of BMAL1 on gut microbiota abundance. These results can introduce new insights into the therapeutic potential of probiotics in AD patients to normalize the abundance of gut microbiota caused by BMAL1. On the other hand, BMAL1 regulators, such as melatonin, can be investigated to evaluate their effects on AD, possibly via regulation of gut flora abundance. Although different pre-clinical and clinical studies have been conducted to study the protective effects of melatonin on the progression of AD, there are no studies that indicate the association between melatonin, circadian rhythm, and gut flora. In addition, this connection may introduce combination therapy with probiotics and melatonin as a suitable and effective therapeutic option for the management of AD. However, more studies are required to prove these claims.

## Conclusion

Based on the mentioned evidence, a hypothesis is provided to explain the association between gut microbiota and circadian rhythm in AD patients. More studies to prove this claim may suggest several other therapeutic interventions to modulate the mentioned communication. For instance, melatonin supplementation and other natural compounds may be considered to modulate this alteration.
